# Insulin-like growth factor-1: a possible marker for emotional and cognitive disturbances, and treatment effectiveness in major depressive disorder

**DOI:** 10.1186/s12991-017-0161-3

**Published:** 2017-10-26

**Authors:** Oleg A. Levada, Alexandra S. Troyan

**Affiliations:** State Institution “Zaporizhzhia Medical Academy of Postgraduate Education Ministry of Health of Ukraine”, 20 Winter boulevard, Zaporizhzhia, 69096 Ukraine

**Keywords:** IGF-1, Growth factors, MDD, Cognitive dysfunction, Depression, Antidepressants

## Abstract

Depression and cognitive dysfunction share a common neuropathological platform. Abnormal neural plasticity in the frontolimbic circuits has been linked to changes in the expression of neurotrophic factors, including IGF-1. These changes may result in clinical abnormalities observed over the course of major depressive disorder (MDD), including cognitive dysfunction. The present review aimed to summarize evidence regarding abnormalities of peripheral IGF-1 in MDD patients and assess a marker and predictive role of the neurotrophin for emotional and cognitive disturbances, and treatment effectiveness. A literature search of the PubMed database was conducted for studies, in which peripheral IGF-1 levels were evaluated. Our analysis revealed four main findings: (1) IGF-1 levels in MDD patients mismatch across the studies, which may arise from various factors, e.g., age, gender, the course of the disease, presence of cognitive impairment, ongoing therapy, or general health conditions; (2) the initial peripheral IGF-1 levels may predict the occurrence of depression in future; (3) peripheral IGF-1 levels may reflect cognitive dysfunction, although the data is limited; (4) it is difficult to evaluate the influence of treatment on IGF-1 levels as there is discrepancy of this growth factor among the studies at baseline, although most of them showed a decrease in IGF-1 levels after treatment.

## Background

Major depressive disorder (MDD) is one of the most prevalent psychiatric diseases [1, 2]. MDD is not only characterized by profound dysregulation of affect and mood, but is also associated with other abnormalities. In recent years, cognitive impairment in major depression has been widely reported. Cognitive dysfunction is a discrete clinical domain [3] subserving functional impairment associated with MDD [4, 5]. Several neuropsychological disturbances, including executive function, attention, memory, processing speed, and psychomotor skills, were found to be during the symptomatic stage of MDD as well as its remission [6–9].

Depression and cognitive dysfunction might share a common neuropathological platform [10]. Neurodegeneration might significantly contribute to the pathogenesis of MDD linked to cognitive complaints. According to this, patients with MDD demonstrate decreased brain volumes in the areas implicated in emotional regulation and cognition, neuronal, and glial death as well as activation of various pathways, which can further to cell death [11]. Existing literature has been focused on putative biological pathways related to cognitive dysfunction in MDD [12]. Recent studies revealed that disturbances in synaptic plasticity, i.e., axon branching, dendritogenesis, and neurogenesis in the prefrontal cortex, hippocampus, amygdala, and ventral striatum may be an important factor in the pathogenesis of MDD and cognitive dysfunction due to MDD [13–17]. Inflammation may also be a contributing factor, as levels of inflammatory cytokines were found to be higher in the prefrontal cortex of patients suffering from depression [11] leading to increased neurodegeneration [18].

Abnormal neural plasticity in the frontolimbic circuits, responsible for emotional and cognitive processing, has been related to changes in the expression of neurotrophic factors, including brain-derived neurotrophic factor (BDNF), neurotrophin-3 (NT-3), neurotrophin-4/5 (NT-4/5), nerve growth factor (NGF), and insulin-like growth factor (IGF-1) [17, 19].

Neurotrophic factors or neurotrophins are a family of proteins, involved in neuronal growth, differentiation, maturation, and survival [17, 20]. They regulate a broad spectrum of brain processes and the equilibrium between neuroregeneration and neurodegeneration [17]. Moreover, these polypeptides might affect synaptic transmission, modulate the activity of various types of neurons or affect memory formation [17].

### Insulin-like growth factor (IGF-1): a brief neurobiological overview

A fundamental analysis of IGF-1 neurobiology has been done earlier by Szczęsny et al. [17]. We have reflected mainly those neurobiological aspects of this neurotrophin, which relate to the mechanisms of development of depression and cognitive dysfunction due to depression.

IGF-1, also called somatomedin C, is a protein, which has a molecular weight of 7.5 kDa. It consists of 70 amino acids and shares 40% identity with insulin [21]. IGF-1 along with IGF-2, insulin, and their respective receptors: IGF-1R, mannose-6 phosphate/IGF-2 (M6P/IGF-2R), IR, hybrid receptor (IR/IGF-1R), and a group of six binding proteins (IGFBP-1–6) compose the Insulin-like Growth Factor family [17, 22].

IGF-1 is synthesized by different organs and its expression is controlled by growth hormone [17]. Liver is the main site of IGF-1 synthesis [22]. In the circulation, over 99% of IGF-1 is bound to IGFBP-1-6 [17]. Although IGF-1 can cross the blood–brain barrier via transcytosis [23], many studies have reported that IGF-1 is also expressed by different cells in the central nervous system [17, 24].

The main function of IGF-1 in the brain is to control cell growth, differentiation, maturation (through mitosis stimulation and DNA synthesis), and metabolic processes (i.e., glucose uptake and protein production) [17]. This agent is also involved in synaptic plasticity by controlling processes of synapses formation, releasing of neurotransmitters, and exciting of neurons [25].

The highest IGF-1 levels are detected in the cerebral structures with extensive vasculature (choroid plexus), as well as in the brain areas with a laminar structure (cerebellar cortex, hippocampus, olfactory bulbs) [17]. IGF-1 receptors are found primarily in synaptic areas [17].

### IGF-1 in the development of depression and cognitive dysfunction: experimental data

Taking into account that IGF-1 can influence many cerebral processes such as synaptic plasticity, adult neurogenesis, and differentiation, it has been assumed that impairments in the IGF-1 system might be responsible for clinical abnormalities observed over the course of MDD, including cognitive dysfunction.

Decreased IGF-1 expression and IGF-1R phosphorylation were revealed in the hippocampus, frontal cortex [26], and olfactory bulb [27] in prenatally stressed rats, which showed depressive-like behavior. After intracerebroventricular administration of IGF-1, these effects in stressed animals were reversed, whilst after concomitant administration of the IGF-1R antagonist—JB1—the effects were completely blocked [27]. Moreover, the administration of antidepressant drugs normalized most of the alterations in the IGF-1 system of the olfactory bulb, which were induced by prenatal stress [27]. These findings demonstrate that prenatal stress changes IGF-1 signaling, which may contribute to the behavioral shifts, observed during MDD [26].

Mutant mice with low serum IGF-1 levels had decreased adult hippocampal neurogenesis together with impaired spatial learning [28]. Administration of exogenous IGF-1 restored the cognitive deficit and adult hippocampal neurogenesis [28]. Reduced expression of IGF-1 along with the decrease in serotonin has been found in male rats with depression-like traits due to social isolation [29].

Disruption of the IGF-1 gene induces neuronal loss in the hippocampus, striatum [30], and dentate gyrus in rats [31] and leads to age-dependent decrease in the differentiation of new cells into neurons [31]. These data indicate that the age-related decrease in the expression of IGF-1 might play an important role in the development of cognitive deficits seen in the elderly. It was shown that such cognitive impairments were reversible by extended systemic administration of IGF-1, pointing that the neurotrophic actions of IGF-1 influence glutamatergic synapses within the hippocampal circuits, thus affecting learning and memory [23].

Immune effects of IGF-1 are also attracting attention, given the importance of neuroinflammation in the pathogenesis of depression and cognitive dysfunction. Inflammation is a significant biological factor that might increase the risk of MDD much more than traditional psychosocial factors. There is strong evidence that depression includes impairments in multiple sides of immunity, which may play a role in the pathophysiology of depressive symptoms [32]. IGF-1 was shown to suppress inflammatory processes by suppressing the expression of inflammatory markers (i.e., IFN-g, IL-1b, TNF-a, iNOS, and GFAP) [33, 34] and enhancing the production of anti-inflammatory agents (IL-4 and IL-10) and BDNF [17, 33].

Despite the presence of harmonized experimental data on the decreased IGF-1 levels in brain in depression and cognitive impairment, there are discrepancies in the estimation of the peripheral IGF-1 concentrations in patients suffering from MDD. This study conducts a narrative review to summarize evidence regarding changes of peripheral IGF-1 in MDD patients and assess a marker and predictive role of the parameters for emotional and cognitive disturbances, and treatment effectiveness.

## Methods

Two independent researchers (Levada and Troyan) conducted the systematic literature search of the PubMed database for the studies published in English from December 1988 to May 2017. If there was inconsistent selection and lack of agreement, a final decision was made through consensus. Search words were: “major depressive disorder”, “unipolar depression”, “cognitive function”, “cognitive dysfunction”, “cognitive deficit”, and “IGF-1”. In the first step, the search results were collected and the titles and abstracts were screened by Levada and Troyan. We included cross-sectional, case–control, longitudinal, and observational studies and reviews, in which peripheral (plasma or serum) IGF-1 levels were evaluated in patients with MDD and in some cases with any depressive disorders. We excluded case-reports or series studies, nonclinical trials and those using samples from tissues other than peripheral blood.

## Results and discussion

In final analysis, we included twenty-three articles that compared different levels of peripheral IGF-1 in patients with depression and healthy controls. There were nine case–control [36, 42, 45, 47, 48, 52–55], two observational [35, 41], five longitudinal [39, 40, 43, 49, 51], and four cross-sectional studies [44–46, 50] and three reviews [17, 37, 38] among them. Eight papers reported comparisons of IGF-1 levels before and after treatment. Two articles compared IGF-1 levels in males and females. Three studies observed a predictive value of IGF-1 levels on occurrence of depression in future. Follow-up periods were 3, 4, and 5 years. Brief descriptions of these studies are presented in Table 1.

**Table 1:** Summary of findings

	Type of study	Measure	Patient	*N*	Age (Mean ± SD)	F %	Depression instrument	MDD severity (Mean ± SD)	IGF-1 in MDD vs controls	Medication and its effects on IGF-1 levels
Bot et al. [35]	Observational	Plasma	MDD/anxiety HC	2112 602	n/a	n/a	n/a	n/a	↑IGF-1 (*p* = 0.006) in AD-free individuals with current disorders; ↑IGF-1 (*p* = 0.09) in AD-free individuals with remitted disorders; ↓IGF-1 (*p* = 0.028) in AD users	Yes, not specified
Rosso et al. [36]	Case–control	Serum	OCD MDD HC	40 37 43	38.7 ± 13.3 42.4 ± 11.9 42.3 ± 11.3	55.0 78.4 65.1	HAMD	6.5 ± 3.0 19.7 ± 2.6	No significant differences. IGF-1 levels correlated to age of MDD onset (*r* = − 0.40, *p* = 0.014)	Yes, citalopram, fluvoxamine, paroxetine, sertraline No changes (*p* = 0.42)
Sharma et al. [37]	A comprehensive review	Serum/plasma	MDD HC	n/a	n/a	n/a	n/a		IGF-1 levels in MDD vs controls were discrepant across studies	n/a
Tu et al. [38]	A meta-analysis and review	Serum/plasma	MDD/BD HC	389 393	n/a	n/a	n/a		↑(*p* < 0.001); Inverse association with MDD duration (*p* = 0.03); IGF-1 may be a marker of cognition	Yes. No change in IGF-1 after Rx (*p* = 0.092)
Chigogora et al. [39]	Longitudinal population-based	Serum	Cohort of adults ≥ 50 years	6017	65.7	55	CES-D8	n/a	↓ and ↑ levels of IGF-1 ↑DD risk; Median levels of IGF-1 ↓DD risk	No
Van Varsseveld et al. [40]	Longitudinal population-based	Serum	MDD No DD	193 995	75.4 ± 6.5	50.3	CES-D	n/a	*M:* as compared to high concentrations, mid concentrations IGF-1 ↓DD probability (OR 0.35); *F:* as compared to high concentrations, low concentrations ↑DD probability (OR 2.66); *F after 3* *year FW:* as compared to high concentrations, mid concentrations ↓DD probability (OR 0.43)	Yes, not specified
Rusch et al. [41]	Observational	Plasma	MDD/PTSD	44	33.3	0	QIDS-SR	13.0	The sleep improved group revealed significant ↓ in MDD (*p* = 0.005) and ↑IGF-1 (*p* = 0.009)	Yes, not specified
Kopzak et al. (2015) [42]	Case–control	Serum	MDD HC	78 92	48.6 ± 13.9 48.1 ± 13.7	44.9 45.7	HAMD	26.4 ± 6.7	↑ (*p* = 3.29E−04)	Yes, not specified. IGF- 1 still ↑ after 6 weeks of Rx (*p* = 0.002)
Krogh et al. [43]	Longitudinal parallel-group	Serum	MDD	79	41.3 ± 12.1	67.1	HAMD	19.0 ± 4.3	Aerobic exercise intervention did not ↑IGF-1 in MDD patients	No
Sievers et al. [44]	Population-based cross-sectional	Serum	West Pomerania Cohort	4079 (1246 DS)	50.0 ± 16.4	51	CID-S	n/a	*F:* ↓IGF-1 ↑DD probability (OR 2.70); *M:* ↑IGF-1 ↑DD probability (OR 3.26)	No
Lin et al. [45]	Cross-sectional	Plasma	Adults aged ≥ 50 years	94	60.7 ± 8.4	58.5	GDS	n/a	*Among older adults with ↓IGF*-*1 levels:* ↑depressive symptoms scores were associated with ↓ of delayed recall and recognition	Yes, not specified
Emeny et al. [46]	Population-based cross-sectional	Serum	KORA-age cohort study	144 DS 841 no DS	*M:*75.4 *F:*75.7	50.0	GDS	n/a	*F:* ↑IGF-1 ↑MDD probability (*p* = 0.045)	No
Szczęsny et al. [17]	A narrative review	Serum/plasma	MDD HC	n/a	n/a	n/a	n/a		Different studies showed an elevation, decrease or no changes in peripheral IGF-1	n/a
Li et al. [47]	Case–control	Serum	MDD HC	15 12	32.3 ± 7.7 31.2 ± 10.2	0.0 0.0	MADRS	n/a	No differences	Escitalopram. No change in IGF-1 after 8 weeks of Rx
Palomino et al. [48]	Case–control	Plasma	BD HC	23 23	27.0 25.7	34.8	HAMD	19.8 ± 8.8	No differences. IGF-1 in schizophrenia correlated with negative symptoms	Yes. No change in IGF-1 levels in BD after 1 year of AP Rx
Weber-Hamann et al. [49]	Longitudinal parallel-group	Serum	MDD (total) Amitriptyline group Paroxetine group	77 34 43	R:51 ± 17 NR:46 ± 16 R:58 ± 16 NR:57 ± 14	72 88.8 62.9 75	HAMD	R:23.9 ± 5.2 NR:22.1 ± 3.9 R:23.0 ± 3.2 NR:23.7 ± 3.5	n/a	Amitriptyline/paroxetine ↓IGF-1 in R (*p* < 0.02)
Rueda Alfaro et al. [50]	Population-based cross-sectional	Plasma	Age > 70 years With DS No DS	100 213	*M*:76.7 ± 5.4 *F*:77.3 ± 6.4	51.11	GDS	n/a	*F:* IGF-1 positively associated with cognition (*p* = 0.04)	No
Michelson et al. [51]	Longitudinal parallel-group	Plasma	MDD (total) on fluoxetine on sertraline on paroxetine	107 37 34 36	40.0 ± 11.4 38.7 ± 14.5 39.9 ± 11.1	75.7 76.5 61.1	HAMD	4.8 ± 2.4 4.7 ± 2.3 4.9 ± 2.8	n/a	Fluoxetine, sertraline, paroxetine. Placebo substitution of paroxetine ↑IGF-1 (*p* = 0.007)
Franz et al. [52]	Case–control	Serum	MDD HC	19 16	34.7 ± 8.8 36.1 ± 6.6	100.0 100.0	HAMD	18.8 ± 3.9	↑(*p* = 0.02)	No
Deuschle et al. [53]	Case–control	Plasma	MDD HC	24 33	47.2 ± 16.4 51.4 ± 19.2	45.8 33.3	HAMD	31.8 ± 5.8	↑(*p* < 0.01)	Fluoxetine, amitriptyline, doxepin. ↓IGF-1 in R (*p* < 0.04)
Michelson et al. [54]	Case–control	Serum	MDD HC	10 10	41.0 ± 8.0 41.0 ± 7.0	100.0 100.0	n/a	n/a	No change (*p* = 0.98)	Yes, not specified
Lesch et al. [55]	Case–control	Plasma	MDD/BD HC	34 34	48.2 ± 12.2 44.7 ± 11.9	67.64 67.64	HAMD	26.9 ± 5.4	↑(*p* < 0.001)	No

*AD* antidepressants; *AP* antipsychotic drugs; *BD* Bipolar disorder; *CES-D* Center for Epidemiologic Studies-Depression Scale; *CES-D8* eight-item Center for Epidemiologic Studies-Depression Scale; *CID-S* WHO WMH-CIDI The Composite International Diagnostic-Screener; *DD* depression disorder; *DS* depressive symptoms; *F* females; *FW* follow-up; *GDS* Geriatric Depression Scale; *HAMD* Hamilton Depression Rating Scale; *HC* healthy controls; *M* males; *MADRS* Montgomery and Asberg Depression Rating Scale; *MDD* major depressive disorder; *n/a* not applicable; *N* number of subjects in the group; *Non-PSD* stroke without depression; *NR* Non-responders; *OR* Odds ratio; *PSD* post-stroke depression; *QIDS-SR* quick inventory of depressive symptomatology 10-item, *R* responders; *Rx* treatment

According to the data, we revealed four main findings: (1) IGF-1 levels in MDD patients mismatch across the studies, which might be due to various confounding factors, e.g., age, gender, the course of the disease, presence of cognitive impairment, ongoing therapy or general health conditions; (2) the initial peripheral IGF-1 levels may predict the occurrence of depression in future; (3) peripheral IGF-1 levels may reflect cognitive dysfunctions; (4) it is difficult to evaluate the influence of treatment on IGF-1 levels as there is discrepancy of this growth factor among the studies at baseline, although most of them showed a decrease in IGF-1 levels after treatment.

The results of current narrative review show that despite divergence the vast majority of studies reported significantly higher levels of peripheral IGF-1 in patients suffering from MDD than in healthy controls. Thus, several case–control studies [35, 42, 52, 53, 55] and one meta-analysis [38] revealed higher levels of peripheral IGF-1 in patients with depression. This positive association between peripheral IGF-1 levels and MDD might indicate a compensatory mechanism to counterbalance the decreased brain IGF-1 expression [35]. In addition, elevation of peripheral IGF-1 concentrations may be explained by the decreased cerebral bioavailability of the neurotrophin due to decreased sensitivity of IGF-1 receptors under the neuroinflammatory stress [38].

Otherwise, decreased levels of peripheral IGF-1 associated with depressive disorders were found only in one epidemiological study and only in women [44], although these discrepancies may arise from the heterogeneities in the depression diagnosis. Most of papers evaluated IGF-1 levels in MDD patients, but Sievers et al. [44] included in their study any depressive disorders, not well specified. This supports mentioned above experimental data that cerebral expression of growth factors is decreased in depression [56].

Controversial findings of IGF-1 levels in MDD patients in comparison with healthy controls, were observed in two recent reviews [17, 38]. Sharma et al. [38] and Szczesny et al. [17] found that studies conducted in different laboratories demonstrated an elevation, decrease or no change in peripheral IGF-1 levels in MDD patients.

Probably the relationship between IGF-1 and depression may have a “U”-shaped pattern [39], which could explain the discrepancies in findings among the studies. In the English Longitudinal Study of Aging, Chigogora et al. observed that lower and higher levels of IGF-1 were related to an elevated risk of depression, whereas the lowest risk was seen around the median levels of the factor [39]. These results are in a partial accordance with the finding that, among women, low IGF-1 levels were associated with higher risk of developing depressive symptoms [44]. Moreover, median levels of IGF-1 lowered the risk of depression disorder [40]. The “U”-shaped pattern of IGF-1-depression relationship is also supported by increased reports of lifetime prevalence of affective disorders in individuals with pituitary dwarfism and acromegaly, which have low and high levels of this hormone, respectively [39]. Hence, both high and low levels of peripheral IGF-1 might be indicators of MDD.

Since the IGF-1—depression association is highly complex, there might be various factors responsible for this. Some studies showed that associations between depression and IGF-1 levels differ across the genders. In the Longitudinal Aging Study Amsterdam (LASA), van Vansseveld et al. [40] found that in males in comparison with high concentrations, mid concentrations decreased the probability of minor depression. Otherwise, in females in comparison with high concentrations, low concentrations increased the probability of MDD. In the Study of Health in Pomerania (SHIP) sex differences were also evident [44]. These differences in the results between genders may be explained by variations of sex hormone or fluctuations of growth hormone and IGF-1-binding protein [40].

Although meta-analysis, Tu et al., found no peculiarities in IGF-1 levels between men and women, they demonstrated that the duration of illness had a significant inverse association with the peripheral IGF-1 levels [38]. Theoretically, a longer duration of illness should be associated with poorer cognitive function in patients with affective disorders and higher peripheral IGF-1 concentrations. However, the inverse association found in the meta-analysis Tu et al. was suggested to be caused by natural decline of IGF-1 expression with aging [38].

Perhaps the expression of IGF-1 is also influenced by biorhythms, particularly sleep disturbances. Rusch and colleagues demonstrated that sleep improvement in MDD patients was related to an increase of the growth factor. At the same time, Krogh et al. found that aerobic exercises (three times per week during 3 months) in patients with mild to moderate MDD had no effect on IGF-1 concentrations [43]. No correlations between baseline IGF-1 levels and clinical features in MDD patients were observed in a case–control study Rosso et al. [36].

Given that depressive mood and cognition are regulated by brain areas that are also responsive for IGF-1 production, it is possible that IGF-1 would modify both depressive and cognitive symptoms [37]. It was hypothesized that increased peripheral IGF-1 levels might not indicate the severity of depression but might indicate the impairment of cognition [38]. Thus, one study found that, among women, IGF-1 was positively correlated with cognitive impairment after adjustment for depression [50]. Similar results were found in patients with schizophrenia [48]. Palomino et al. demonstrated a positive association between peripheral IGF-1 concentration and the scores of the PANNS scale, which assesses cognitive deficits in schizophrenia; therefore, this positive correlation suggested that elevated levels of the neurotrophin might parallel cognitive impairment [48]. On contrary, in elderly people, decreased IGF-1 levels were significantly associated with the decline of cognitive functioning and higher prevalence of depressive symptoms [45], whereas higher levels of IGF-1 were seen in those with improved cognitions [48].

Whether peripheral IGF-1 levels may predict the probability of depression was investigated in three studies [39, 40, 44]. In the previously mentioned SHIP-study, which included 4079 subjects [44], low IGF-1 levels in females and high IGF-1 levels in males predicted the incidence of depressive disorder in the general population after a 5-year follow-up period. In addition, in the LASA, which included 1188 elderly participants (older than 65 years), mid concentrations of IGF-1 levels in women, as compared to high concentrations, decreased the probability of minor depression, while no prospective associations were detected in men after a three-year follow-up period [40]. Furthermore, Chigogora et al. reported a “U”-shaped pattern of MDD risk in both genders after 4 years of follow-up in subjects initially free of depression symptoms [44].

The influence of drug treatment on IGF-1 levels was also examined in MDD patients. It was suggested that antidepressant treatment modifies the relationship between depression and plasma IGF-1 level [35]. Weber-Hamann et al. and Deuschle et al. found that antidepressant treatment (amitriptyline, doxepin, fluoxetine, paroxetine) was related to a significant decline of plasma IGF-1 levels [49, 53]. Moreover, it was reported that only patients in remission had decreased IGF-1 concentrations after drug treatment [49, 53]. At the same time, antidepressant-free patients in remission had a trend towards higher IGF-1 levels [35]. Furthermore, it was demonstrated that the placebo substitution of paroxetine resulted in a significant increase in IGF-1 plasma levels [37, 51]. On contrary, a meta-analysis, Tu et al. [38,] and several case–control studies [36, 42, 47, 48] revealed no significant changes in peripheral IGF-1 levels after drug treatment.

## Limitations

Discussed studies did not take into account the possibility of intra-individual variability in measurements of IGF-1 levels of the participants, which may arise from the lack of standardization of performing IGF-1 assay [57] and from normal biological variations, which may lead to false IGF-1 elevations [58]. In addition, to our knowledge, there are no studies that simultaneously compared peripheral and central levels of IGF-1 in MDD patients. Therefore, future studies should control for standardized assays for measuring IGF-1 levels, have repeated assessments, and elucidate the relationship between peripheral and central nervous system levels of this factor in MDD patients and healthy controls.

## Conclusions

From the current review, we can conclude that there are discrepancies in IGF-1 levels in MDD patients across the studies, although the majority demonstrates higher levels of peripheral IGF-1 compared to healthy controls. These discrepancies may arise from various factors, such as gender, age of onset, the course of the disease, ongoing therapy, cognitive deficit, and general health conditions. The relationship between IGF-1 and cognitions needs further investigation, as it might be a useful biomarker for cognitive disturbances. Moreover, peripheral IGF-1 levels may play a predictive role in the occurrence of depression, thus low IGF-1 levels in women and high in men predict the development of MDD in general population. Some studies show that antidepressant treatment decreases IGF-1 levels.

## Abbreviation

MDD: major depressive disorder
